# Combined neuromuscular electrical stimulation with motor control exercise can improve lumbar multifidus activation in individuals with recurrent low back pain

**DOI:** 10.1038/s41598-021-94402-2

**Published:** 2021-07-20

**Authors:** Sranya Songjaroen, Panakorn Sungnak, Pagamas Piriyaprasarth, Hsing-Kuo Wang, James J. Laskin, Peemongkon Wattananon

**Affiliations:** 1 Biomechanics and Sport Lab, Faculty of Physical Therapy, Mahidol University, 999 Phuttamonthon 4 Road, Salaya, Nakhon Pathom 73170 Thailand; 2 Motor Control and Neural Plasticity Lab, Faculty of Physical Therapy, Mahidol University, 999 Phuttamonthon 4 Road, Salaya, Nakhon Pathom 73170 Thailand; 3 Sports Physiotherapy Lab, School and Graduate Institute of Physical Therapy, College of Medicine, National Taiwan University, No. 17, Xuzhou Rd., Zhongzheng District, Taipei City, 100 Taiwan; 4 School of Physical Therapy and Rehabilitation Science, University of Montana, 135 Skaggs Building, Missoula, MT 59812 USA

**Keywords:** Rehabilitation, Pain management

## Abstract

Motor control exercise (MCE) is commonly prescribed for patients with low back pain. Although MCE can improve clinical outcomes, lumbar multifidus muscle (LM) activation remains unchanged. Neuromuscular electrical stimulation (NMES) can be used to re-activate motor units prior to MCE which should result in increased LM activation. Therefore, this study aimed to explore the immediate effects of NMES combined with MCE on LM activation and motor performance. Twenty-five participants without low back pain (NoLBP) and 35 participants with movement control impairment (MCI) were recruited. Participants with MCI were further randomized to combined NMES with MCE (COMB) or sham-NMES with MCE (MCE) group. Ultrasound imaging was used to measure LM thickness at rest, maximum voluntary isometric contraction (MVIC), and NMES with MVIC. These data were used to calculate LM activation. Quadruped rocking backward was used to represent motor performance. LM activation and motor performance were measured at baseline and after one-session of intervention. Results showed that both COMB and MCE groups had significantly lower (*P* < 0.05) LM activation compared with NoLBP group at baseline. Additionally, both COMB and MCE groups demonstrated significant improvement (*P* < 0.05) in motor performance while COMB group demonstrated significantly greater improvement (*P* < 0.05) in LM activation compared with MCE group. Individuals with MCI still have persisting LM activation deficit. Our key findings suggest that combined NMES and MCE may have better ability to improve LM activation in individuals with MCI. These findings would support the utility of NMES to induce a priming effect before MCE.

## Introduction

Existing lumbar multifidus muscle (LM) activation deficit is one factor that can cause recurrent low back pain (rLBP)^1,2^. The primary function of the LM is to provide spinal stability during static and dynamic tasks^1,3,4^. The LM activation deficit would compromise the stabilizing system, thereby increasing the risk of re-injury in those with rLBP^1,3,4^. Evidence demonstrates asymptomatic individuals with history of low back pain still have the LM activation deficit^1,4–7^. Prior studies using surface-electromyography (sEMG) have demonstrated reduced LM activity in individuals with rLBP suggesting an incapability to recruit all LM motor units during maximal voluntary contraction^8,9^. Therefore, restoration of LM activation in those with low back pain is necessary to prevent recurrent symptoms.

Arthrogenic muscle inhibition has been proposed as one potential mechanism that causes deficits in LM activation^1,4^. This reflex inhibition is a built-in protective mechanism that occurs after injury^1,4^. It interferes the transmission of the sensory and motor signals between brain and injured structures that further inhibits individual with LBP to fully recruit motor unit available in the muscle^4^. This LM activation deficit subsequently causes inadequate LM contraction to stabilize the lumbar spine^10^. Lack of lumbar stability will result in increased shear force during prolonged mal-alignment posture or repeated lumbar movement leading to soft tissue injury^10,11^. In addition, persisting muscle activation deficit may further cause neuromuscular control maladaptation, particularly in movement control impairment (MCI) subgroup^12–15^. One preliminary study demonstrated that this subgroup still has LM activation deficit^15^. Accordingly, physical therapy intervention should be designed to address the LM activation deficit to restore muscle function.

Motor control exercise (MCE) is one of the treatments that physical therapists commonly provide to patients with MCI to restore LM function^16^. Although several studies demonstrated significant short-term improvement in clinical outcomes in patients with rLBP after MCE, these improved outcomes were not superior to other forms of exercise, and no long-term effectiveness has been reported^16,17^. One study demonstrated no significant change in LM thickness after MCE even though the results showed improvement in pain and disability^18^. This could be postulated that MCE alone might not be sufficient to enhance motor unit recruitment, particularly those in MCI subgroup.

Neuromuscular electrical stimulation (NMES) is one potential intervention that can restore LM activation^19–21^. Although evidence indicates significant improvement in LM activation after receiving NMES^19–21^, this is a passive intervention that may not guarantee the long-term normalization of LM activation. Accordingly, individuals with MCI should be actively trained the LM in conjunction with NMES to elicit the improvement in LM activation.

There is a need to investigate whether the intervention program can improve muscle activation after one session. Therefore, the overall objective of this study was to explore the immediate effects of combined NMES and MCE on LM activation and motor performance in individuals with MCI. We first compared the LM activation between individuals without LBP (NoLBP) and individuals with MCI who received combined NMES with MCE (COMB) or combined sham-NMES with MCE (MCE). We hypothesized that those with MCI (COMB and MCE) would have lower LM activation than NoLBP. Then, we explored the immediate effect of NMES combined with MCE on LM activation and motor performance. We hypothesized that both COMB and MCE groups would show improvement in motor performance, but COMB group would demonstrate a greater improvement in LM activation than the MCE group. Finally, we wanted to explore the relationship between LM activation and motor performance, in which we hypothesized that LM activation would be correlated with motor performance.

## Methods

### Study design

A prospective randomized controlled trial with assessor, participant, and therapist blinded to the intervention assignment was used to determine the immediate effects of combined NMES and MCE on LM activation and motor performance in MCI subgroup of rLBP. The funders played no role in the design, conduct, or reporting of this study.

### Participants

This study used a sample of convenience. Sixty participants (35 rLBP and 25 NoLBP) were recruited from two University physical therapy clinics. The inclusion criteria were (1) aged between 20 and 40 years, (2) having a recurrent episode of LBP and at least two episodes interfered with activities of daily living (e.g., pain during driving a car, prolonged sitting, etc.) or required treatment (e.g., have to take a pain medication, visit physical therapist, etc.) and (3) having clinical lumbar instability (presence of aberrant movement during active forward bend and passive straight leg raising greater than 91 degrees)^22,23^.

We selected the age ranged between 20 and 40 years because previous studies demonstrated that patients with LBP younger than 40 years of age were more likely to have MCI and would benefit from MCE, while patients older than 40 years old were more likely to have a specific low back condition, such as degenerative spine, spondylosis, or spinal stenosis^22,23^. Age less than 40 years, a positive aberrant movement pattern test and a passive SLR greater than 91 degrees are indicators for classifying patients with LBP into MCI subgroup^22,23^. Evidence suggests that if these three criteria have been met, the positive likelihood ratio would increase up to 4.0 indicating that patients with LBP would have 4 times more likely to have MCI^22^.

The exclusion criteria were (1) clinical signs of systemic disease, (2) definitive neurological signs including pain, weakness, or numbness in the lower extremity, (3) previous spinal surgery, (4) diagnosed osteoporosis, severe spinal stenosis, or inflammatory joint disease, (5) body mass index greater than 30 kg/m^2^, (6) active treatment of another medical illness that would preclude participation in any aspect of the study, and (7) limited hip mobility or other hip problems that may affect MCE.

This study was a part of ongoing project. Our pilot study demonstrated improvement in LM activation with large effect size (Cohen’s d_z_ = 0.96). We used this effect size to calculate sample size. The total sample size of 9 participants per group was required to detect effect size of 0.96 at level of confidence of 0.05 and 80% power. Therefore, our sample size of 60 would be sufficient. Data were collected between June 2019 and March 2020.

### Instruments

Rehabilitative ultrasound imaging (RUSI; model CX50, Philips, NV, USA) with a broadband linear array (model L12-3) transducer was used to measure LM thickness at L4-5 facet joint during resting, MVIC, and combined NMES with MVIC. LM thickness change can be used to represented muscle activation^2,24,25^. Our pilot work demonstrated LM thickness was linearly correlated with muscle activity using electromyography in different levels of LM activation. Therefore, the LM thickness can be alternatively used to represent the level of LM activation.

Universal goniometer (BASELINE®, NY, USA) was used to measure knee flexion angle during quadruped rocking backward to represent motor performance. Previous study demonstrated the concurrent validity of the universal goniometer using digital inclinometer as a reference standard ^26^.

### Measurement

#### Lumbar multifidus muscle thickness

**Figure 1:**
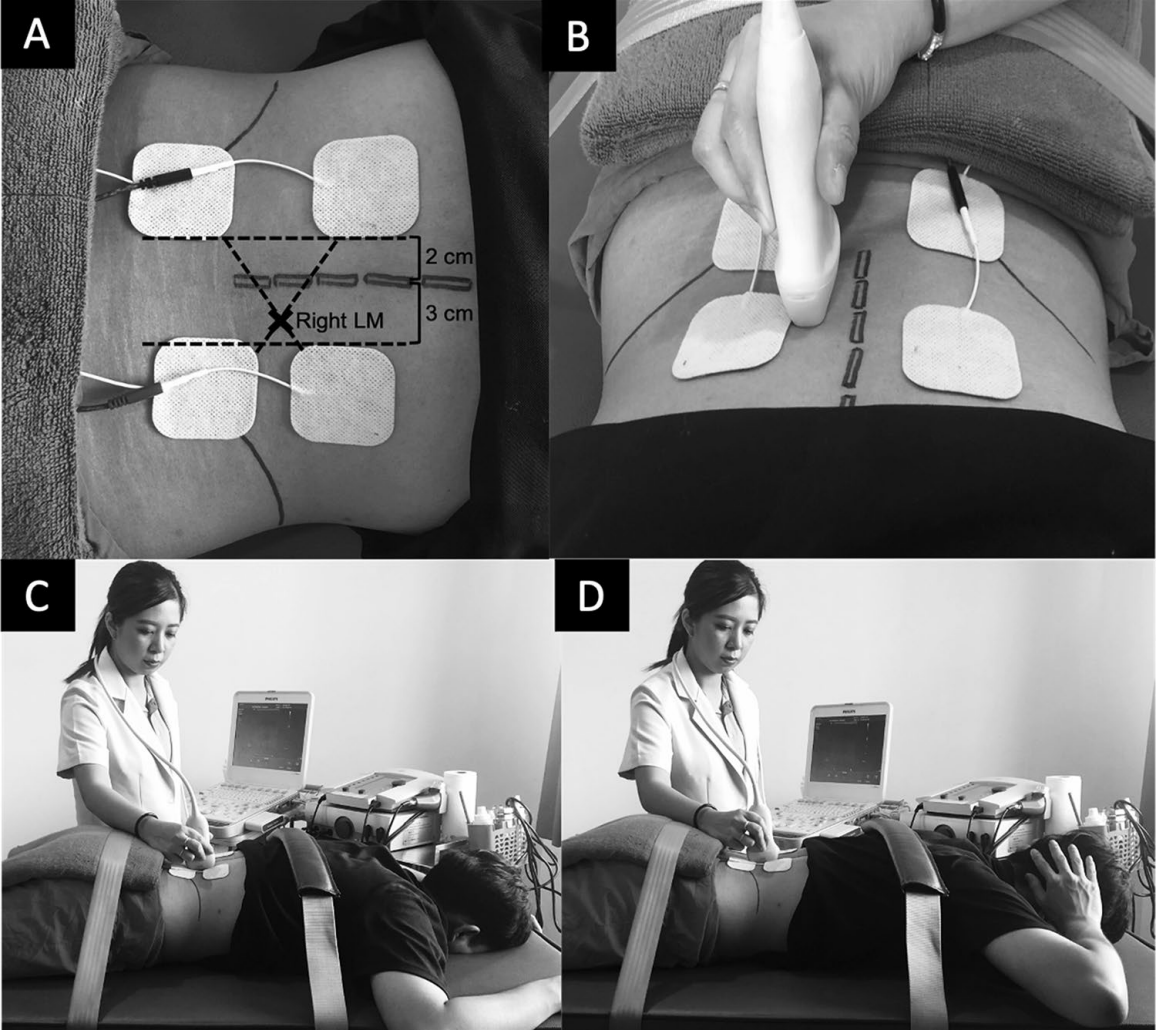
Neuromuscular electrical stimulation placement to stimulate right lumbar multifidus muscle (**A**) and ultrasound transducer placement at right L4-5 facet joint (**B**) during resting (**C**) and maximum voluntary isometric contraction with and without stimulation (**D**).

The participant was placed in prone with the thorax (T3 level) and pelvis (S2 level) securely strapped to the treatment bed. Towel roll was placed under the pelvis and abdomen if needed to keep lumbopelvic in neutral position. The body landmarks (Fig. 1A) including lumbar spinous process of L1-L5, L4-5 facet joint (2 cm lateral to lower half of spinous process of L4), and NMES electrode placement (the first two point at 3 cm lateral to the L3 and L5 spinous processes on tested side and another two points at 2 cm away from spinous processes on the opposite side that cause two diagonal lines intersecting at the LM) were identified^15^. The L4-5 facet joint landmark was used to place the RUSI transducer to measure LM thickness (Fig. 1B). The LM thickness was the distance between tip of L4-5 facet joint and thoracolumbar fascia.

The researcher captured two RUSI images of right LM thickness at rest (LM_REST_) during terminal expiration (Fig. 1C). Then, the participant was asked to perform two repetitions of MVIC (Fig. 1D) with one-minute rest between repetitions, while the researcher simultaneously recorded right LM thickness data (LM_MVIC_). The NMES (Sonopuls-490 combination therapy, Enraf–Nonius BV, Netherlands) with 5 × 5 hydrogel surface electrodes were used to stimulate right LM. The NMES was set using interferential mode at 6000 Hz, amplitude modulated 20–50 Hz with scanning mode^15^. These settings aimed to minimize the effect of nerve accommodation and provide electrical signals similar to those observed during a normal voluntary contraction^27–29^. The intensity was increased until maximum pain tolerance. At this point, the participant was asked to perform another two MVIC with one-minute rest between repetitions, whereas the researcher recorded right LM thickness data (LM_NMES+MVIC_). A five-minute rest was provided to minimize muscle fatigue. After that, the researcher performed the same protocol to measure left LM thickness. Our previous pilot study demonstrated no order effect whether we measured right or left first. In addition, our pilot work demonstrated excellent intra- and inter-rater reliability (ICC_3,1_ = 0.91–0.99 and ICC_2,2_ = 0.95, respectively) for LM thickness measurement.

#### Motor performance

**Figure 2:**
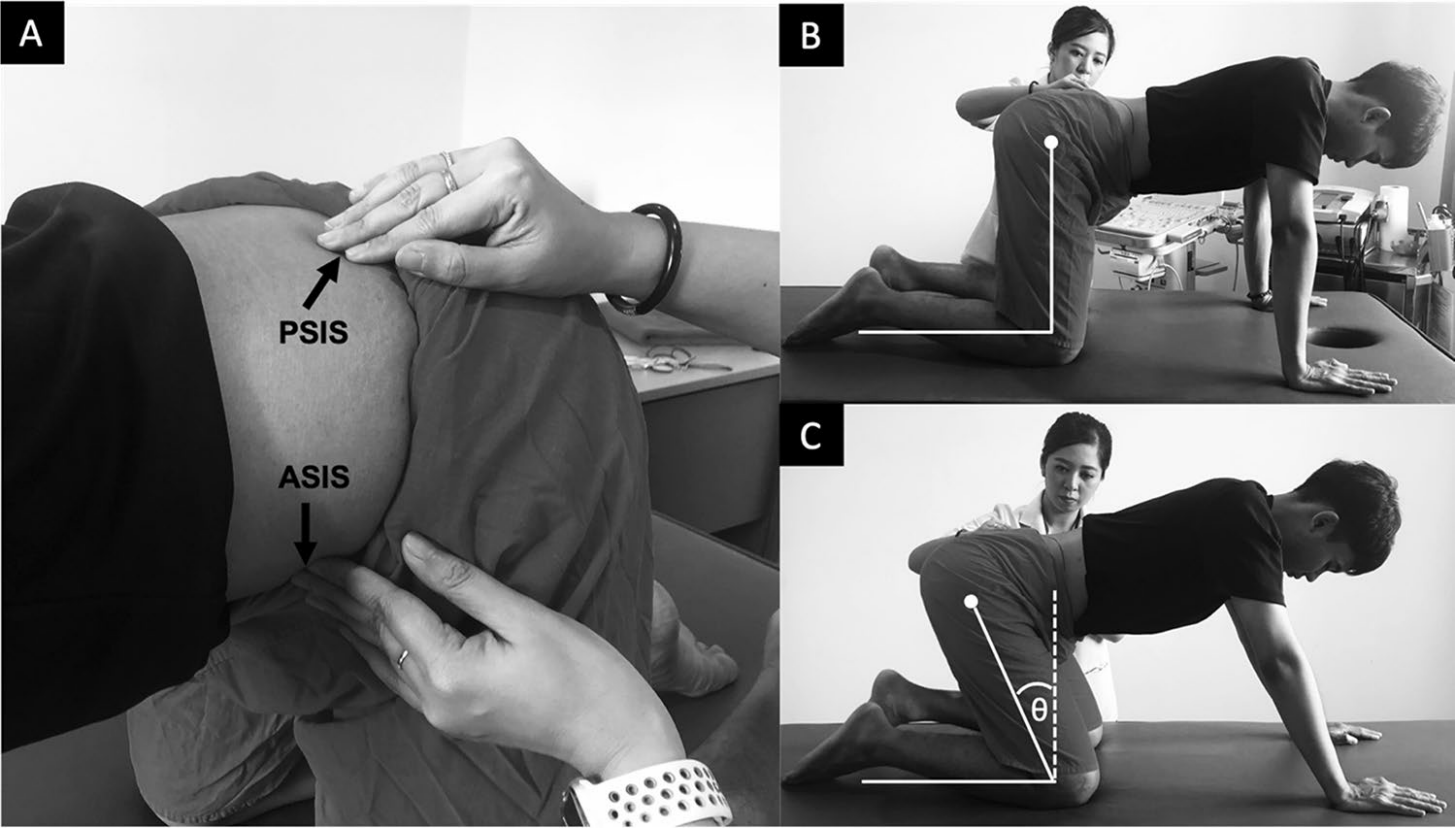
Hand placement at anterior superior iliac spine (ASIS) and posterior superior iliac spine (PSIS) to monitor pelvic position (**A**) during quadruped rocking backward from starting position (**B**) until the pelvis starts to move posteriorly (**C**) where knee flexion angle (θ) used to represent motor performance.

The participant was asked to assume the quadruped position, both hands and knees were symmetrically placed a shoulder or hip width apart with the knees in 90-degree of flexion. The researcher used both hands to palpate ASIS and PSIS (Fig. 2A) and move the lumbar spine and the pelvis in neutral position as a starting position (Fig. 2B). The researcher instructed the participant to maintain this neutral position and gently rock backward as far as they could until the researcher noted the pelvis starting to move posteriorly (Fig. 2C). In this, rocked backward position, the researcher measured the change in knee flexion angle. This angle represented motor performance (greater knee flexion angle means greater ability to control the LM. Our preliminary study had established a minimal detectable change of motor performance as 7 degrees.

### Procedure

The study protocol was approved by the university institutional review board (COA. No. 2018/215.0712) and registered in clinical trials (ClinicalTrials.gov ID: NCT03786627; 20/12/2018). The researchers informed all participants of the purposes and procedures of the study. The participants provided their written informed consent before data collection. This human research followed the principles of the Declaration of Helsiki. Informed consent for publication of identifying information/images in an online open-access publication has also been obtained.

**Figure 3:**
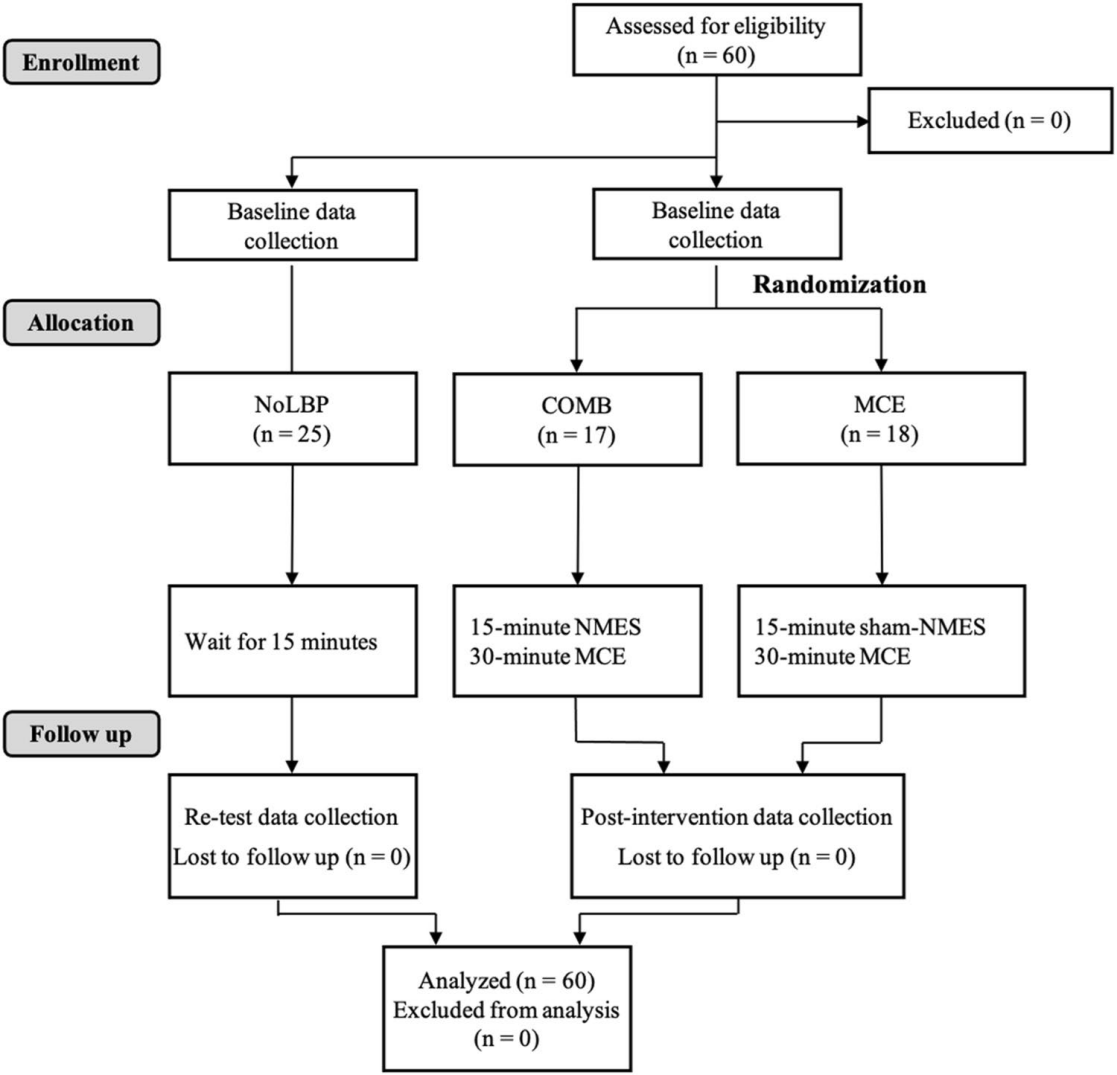
CONSORT flow chart. *Note*: NoLBP = no low back pain group, COMB = combined neuromuscular electrical stimulation (NMES) with motor control exercise group, MCE = sham-NMES and motor control exercise group.

All participants were asked to fill out the demographic data form, while participants with rLBP were asked to further provide rLBP behavior. The researcher who was blinded to the groups performed all of the data collection. First, the researcher measured LM_REST_, LM_MVIC_, and LM_NMES+MVIC_ of right and left LM thickness. Then, the researcher measured motor performance during quadruped rocking backward. Once this baseline data was collected the blinded researcher left the room to preserve their blindness. The participants in NoLBP group were asked to rest for 15 min, while participants with rLBP were randomly assigned to either combined NMES with MCE (COMB), or MCE (MCE) using randomly generated number in an opaque envelope. The COMB group received 15 min of NMES followed by 30-min MCE, whereas the MCE group received 15 min of sham-NMES followed by 30-min MCE. Sham-NMES was used to blind the participant and the therapist. The NMES was operated by another researcher who did not involve in any aspect of data collection. After intervention, the researcher who blinded to the group entered the room again and performed post-intervention data collection. All RUSI data were exported for further data analysis. The CONSORT flow chart of this study is provided in Fig. 3.

### Intervention

The COMB group received 15-min NMES to stimulate the LM. The NMES electrodes were placed at 3 cm lateral to L3 and L5 levels bilaterally. The NMES parameters were set as testing protocol^27–29^. The NMES intensity was set at the level that elicited palpable contraction. Our preliminary work showed an excellent correlation (r = 0.98) between palpation and observation of muscle thickness change using the RUSI. The MCE group was set up in an identical fashion, but the intensity was set at zero.

A quadruped rocking backward was used for MCE. The participant performed MCE for 30 min (15 repetitions per set for 8–12 sets depending on their own performance). One-minute rest was provided between sets to minimize muscle fatigue. To ensure that the participant performed exercise correctly, the therapist palpated ASIS and PSIS, and provided verbal feedback during training (Fig. 2A).

### Data analysis

A MATLAB program version R2014a (The MatWorks Inc, MA, USA) was used for data analysis. The LM thickness during resting, MVIC, and combined NMES with MVIC were used to calculate percentage of LM activation (LM_ACT_) using following equation^15^.

$${LM}_{ACT}=\frac{{LM}_{MVIC}-{LM}_{REST}}{{LM}_{NMES+MVIC}-{LM}_{REST}}X 100$$

Prior to main data analysis, previous studies demonstrated that impairment in the LM including LM activation deficit was presented bilaterally in patients with chronic and recurrent LBP^1,7,30^. To ensure that no significant difference between painful and non-painful sides, we selected participants who had a history of unilateral LBP and transformed into ipsilateral and contralateral sides before performing statistical analysis. We found that no significant side-to-side difference (*P* > 0.05) in participants with unilateral pain; therefore, we used the average value of LM_ACT_ for our main analysis.

The LM_ACT_ from NoLBP group at baseline were used to determine intra-session test–retest reliability of our protocol and establish 95% confidence minimal detectable change (MDC_95_). Our data demonstrated excellent intra-session test–retest reliability for right and left LM_ACT_ (ICC_3,1_ = 0.91 and 0.95, respectively), while MDC_95_ were 5.38 and 2.46, respectively. We used MDC_95_ of 5.38 as a conservative approach in addition to statistical analysis to determine true change beyond the measurement error.

Knee flexion angle, which represented motor performance, was used to determine the effect of combined NMES with MCE. In addition, motor performance and LM activation from COMB and MCE groups at baseline were used to determine the correlation between motor performance and LM activation.

### Statistical analysis

Statistical analyses were performed using SPSS program version 21 (IBM Corp., NY, USA). Shapiro–Wilk test was used to determine normality. All continuous parameters were normally distributed, except LM_ACT_ at baseline and re-test for NoLBP group. One-way ANOVA was used to compare age and BMI among groups, while an independent t-test was used to compare LBP behavior between COMB and MCE groups at baseline. A chi-square test was used to determine the difference in sex proportion among groups.

For main analysis, Kruskal–Wallis test was used to compare LM_ACT_ among groups at baseline and post-intervention. A Mann–Whitney U test was used as a post-hoc pairwise comparison between groups when Kruskal–Wallis test demonstrated significant difference among groups. A Wilcoxon’s test was used to determine difference between baseline and post-intervention for COMB and MCE groups and between baseline and re-test for NoLBP group.

To specifically determine the effect of combined NMES and MCE on LM activation and motor performance, a two-way mixed ANOVA with post-hoc Bonferroni correction was used to compare LM_ACT_ between COMB and MCE groups at baseline and post-intervention, as well as within group between baseline and post-intervention for each group. In addition, an independent t-test was used to determine the difference in improvement between groups. Effect size was also calculated. A Pearson’s correlation test was used to determine the relationship between LM_ACT_ and motor performance for participants with MCI. Significant level was held at 0.05 for all statistical analyses.

## Results

Table 1 demonstrates demographic data for all participants and LBP behavior for those participants with MCI. Statistical analyses showed no significant difference (*P* > 0.05) in demographic data among three groups, and no significant difference (*P* > 0.05) in LBP behavior between COMB and MCE groups.

**Table 1:** Demographic data and LBP behavior.

Parameter	NoLBP Mean (SD)	COMB Mean (SD)	MCE Mean (SD)
Age (years)	22.2 (2.3)	24.3 (4.9)	24.8 (5.0)
Sex (%female)	60.0	64.7	44.4
BMI (kg/m^2^)	22.0 (1.6)	21.3 (3.4)	20.8 (2.6)
Duration (months)	N/A	48.6 (57.9)	46.2 (28.3)
Frequency per year	N/A	13.4 (15.8)	10.6 (13.5)
Time since last episode (days)	N/A	23.0 (25.7)	21.8 (18.2)
Duration for last episode (days)	N/A	3.1 (3.3)	7.0 (17.1)
Pain at last episode (0 = no pain, 10 = intolerable pain)	N/A	4.5 (1.7)	5.2 (1.4)
Disability at last episode (0 = no disability, 10 = total disability)	N/A	3.1 (1.7)	4.2 (1.9)

NoLBP = no low back pain group, COMB = combined neuromuscular electrical stimulation (NMES) with motor control exercise group, MCE = sham-NMES and motor control exercise group, BMI = body mass index.

**Figure 4:**
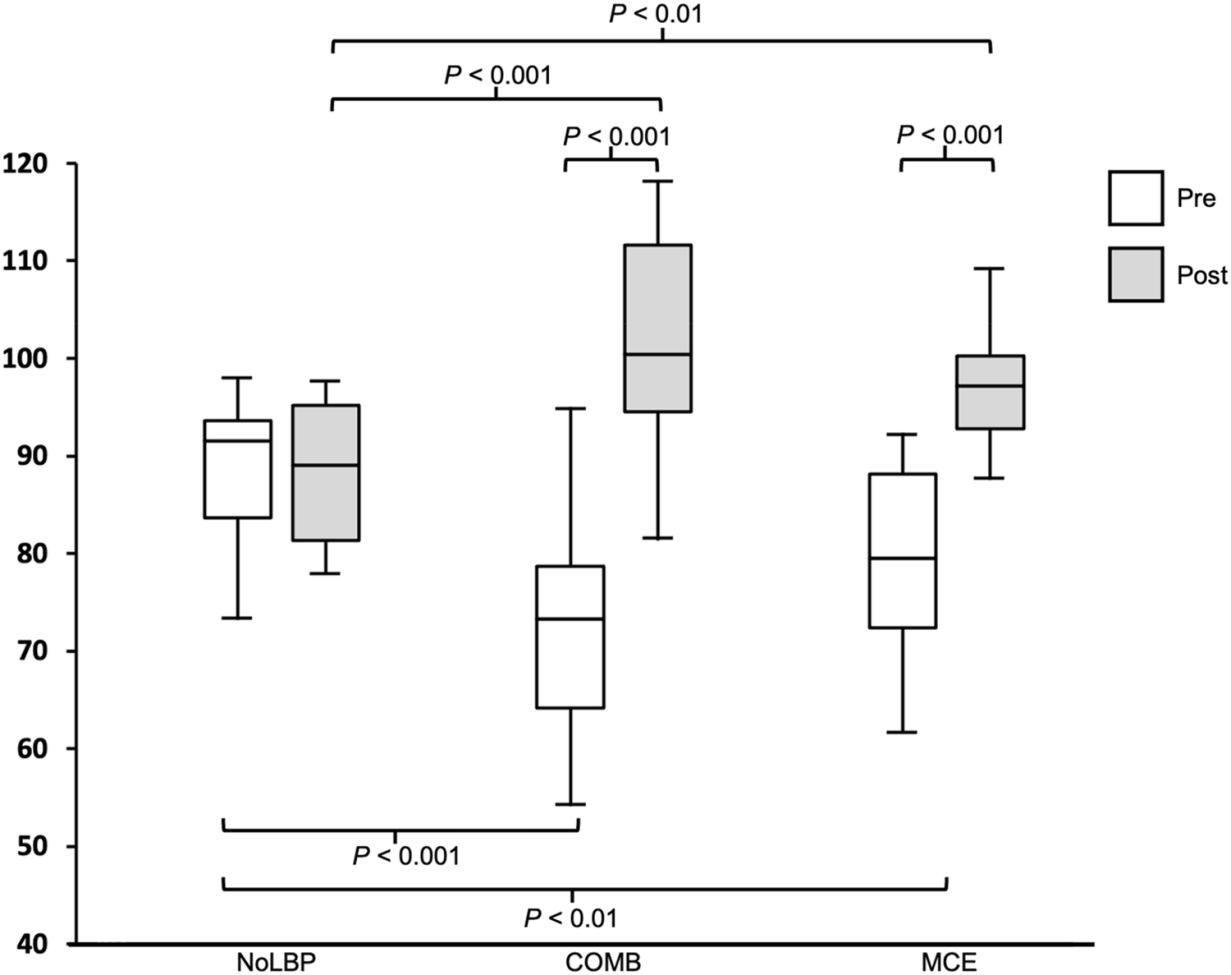
Lumbar multifidus muscle activation pairwise between-group comparisons among no low back pain (NoLBP), combined neuromuscular electrical stimulation with motor control exercise group (COMB), and motor control exercise group (MCE), as well as within-group comparison between baseline and post-intervention for each group.

Kruskal–Wallis test demonstrated a significant difference (*P* < 0.01) in LM_ACT_ among groups at baseline and post-intervention. Post-hoc pairwise comparisons showed both COMB and MCE groups had a significantly lower (*P* < 0.01) LM_ACT_ compared with the NoLBP group at baseline, while the LM_ACT_ in COMB and MCE groups was significantly greater (*P* < 0.01) than the NoLBP group post intervention (Fig. 4). Wilcoxon’s test revealed a significant increase (*P* < 0.001) in the LM_ACT_ in both the COMB and MCE groups between baseline and post-intervention, while no significant change (*P* > 0.05) was observed in the NoLBP group (Fig. 4).

A two-way mixed ANOVA (Table 2) demonstrated a significant main effect of time (*P* < 0.05). Although post-hoc pairwise comparisons demonstrated significant improvements (*P* < 0.05) in LM activation and motor performance for both COMB and MCE groups, no significant difference (*P* > 0.05) was detected between these groups at baseline and post-intervention. However, an independent t-test demonstrated that the COMB group had a significantly greater improvement (*P* < 0.05) than their MCE group counterparts when changes in LM activation were compared. No significant differences (*P* > 0.05) were detected when comparing the baseline to post measurements of motor performance between groups. A Pearson’s correlation test demonstrated a significant positive correlation (r = 0.43, *P* < 0.05) between LM activation and motor performance.

**Table 2:** Lumbar multifidus muscle activation pairwise between-group comparisons between combined neuromuscular electrical stimulation with motor control exercise group (COMB), and motor control exercise group (MCE), as well as within-group comparison between baseline and post-intervention for each group.

Parameter	Time	Group		Group Difference	p-value	ES (Cohen’s d)
		COMB Mean (SD)	MCE Mean (SD)			
LM activation	PRE	70.2 (15.2)	77.4 (13.5)	7.2	0.15	0.50
	POST	100.4 (14.7)	97.3 (6.3)	3.1	0.43	0.27
	Change	30.2*	19.9*	10.3*	< 0.05	0.70
	p-value	< 0.001	< 0.001			
	ES (Cohen’s d_z_)	1.98	1.43			
Motor performance (degrees)	PRE	18.5 (7.4)	22.8 (7.1)	4.3	0.09	0.59
	POST	35.6 (7.5)	36.3 (7.8)	0.7	0.79	0.09
	Change	17.1*	13.5*	3.6	0.10	0.58
	p-value	< 0.001	< 0.001			
	ES (Cohen’s d_z_)	2.47	2.31			

*Exceeding 95% confidence minimal detectable change.

## Discussion

This study was designed to explore the immediate effects of NMES combined with MCE on LM activation and motor performance in participants with rLBP suspected to have MCI. The results support our hypothesis in that both COMB and MCE groups demonstrated significantly lower LM activation compared with NoLBP group. This finding also supports the existence of LM activation deficits in MCI subgroup of rLBP and is consistent with the previous studies reporting persisting LM activation deficits which do not automatically recover after an episode of LBP^1,4,6,7^. This LM activation deficit may cause the insufficient forces required to stabilize the lumbar spine during daily activities, thereby increasing the risk of recurrent episode of low back symptoms^1,9,11^.

Our main findings partially support our hypothesis in which we found both COMB and MCE groups demonstrated significant improvement in motor performance, and COMB group demonstrated greater improvement in LM activation compared with MCE group. These findings suggest that a combined treatment of NMES and MCE has an enhanced ability to improve LM activation. We used the NMES to stimulate inactive motor units in the LM, and then trained the participants to voluntarily recruit those motor units via the quadruped backward rocking activity. Previous studies support this finding in that the NMES has a potential to facilitate motor unit recruitment in the deep back muscles^19–21^, thereby increasing muscle thickness as measured by RUSI. Previous researchers suggest that the NMES has the ability to initiate the contraction of innervated LM^31^. Although those studies indicate the usefulness of the NMES^19–21^, it was a passive treatment without the added voluntary control. Therefore, we used NMES for priming effect followed by MCE to restore the ability to voluntarily recruit those motor units in the LM.

A study demonstrated that MCE alone cannot change the LM thickness following 6-week exercise program even though the patients demonstrated clinical improvement in pain and disability^18^. Those findings suggest that MCE might not be able to help the individual to recruit greater number of motor units. Our findings indicate that the NMES may be required to re-activate motor units prior to MCE to regain the ability to recruit motor units in the LM.

Although we expected that LM activation in MCE group would remain unchanged, our result demonstrates increase in LM activation after just one-session of exercise program. This finding is not consistent with prior study findings^18^. This different finding could be a result of the discrepancy in the motor control training programs between their study and ours. Their study focused on the co-contraction of transverse abdominis muscle and LM during lower limb movement which was considered as a low-intensity exercise^1,16^, while our training was focusing on maintaining the contraction of the LM during a quadruped rocking backward exercise. Thus, our MCE may have induced greater neuromuscular responses, including enhanced motor unit recruitment, resulting in increased LM activation.

In addition, a recent randomized controlled trial demonstrated no additional clinical benefit when supplementing NMES with MCE in patients with chronic LBP after a 6-week intervention program^32^. This finding could be a result from their intervention, the NMES was applied to the paraspinal muscles, while the MCE was focused on abdominal bracing with lower limb movements. However, our intervention was specific to induce a priming effect with the NMES prior to the MCE^19,21,31^. Another potential explanation was that our study investigated a MCI subgroup of rLBP who was more likely to have a LM activation deficit as the underlying cause of LBP^12–15^.

Finally, we also explored the correlation between LM activation and motor performance as a result of the quadruped rocking backward exercise. Our finding was consistent with our hypothesis in which individuals with a greater LM activation would have greater ability to maintain their lumbar spine and pelvis in a neutral position; represented by a greater knee flexion angle at the end of the movement^11^. This finding suggests the important role of the LM to stabilize the lumbopelvic region during movement^1,4^.

## Limitations

Some limitations should be addressed in this current study. First, this study recruited individuals with underlying MCI, which would limit the generalizability of our findings to general LBP population. Another limitation is hip muscle tightness, which would potentially affect MCE in quadruped rocking backward position. We did not assess the presence of muscle tightness in this study; however, we did screen our participants for limited hip mobility or other hip problems that could affect MCE as one of exclusion criteria. Further comprehensive intervention study should take this issue into consideration. In addition, future study should select the specific measurement to assess lumbar stability as we believe that increased LM activation would improve spinal stability.

## Data Availability

The datasets used and/or analyzed during this study would be available from corresponding author upon reasonable request.
